# Intestinal microbiota alteration in women with adenomyosis and clinical characteristics correlation

**DOI:** 10.3389/fmicb.2026.1773822

**Published:** 2026-08-06

**Authors:** Yujing Guo, Hua Duan, Fang Chen, Yanan Chang, Yuanyuan An

**Affiliations:** Department of Minimally Invasive Gynecology, Beijing Obstetrics and Gynecology Hospital, Beijing Maternal and Child Health Care Hospital, Capital Medical University, Beijing, China

**Keywords:** adenomyosis, biomarker, clinical correlation, functional prediction, gut microbiota

## Abstract

**Introduction:**

Adenomyosis is an enigmatic disease with estrogen dependence, and the exact pathogenic mechanisms remain confusing. Limited evidence points out the presence of gut flora changes in adenomyosis. However, evidence on how adenomyosis and gut microbiota crosstalk remains insufficient.

**Methods:**

This study included 30 female diagnosed with adenomyosis and 28 healthy control participants. Fecal samples were collected from all subjects prior to the initiation of pharmacological treatment. The 16S rRNA gene sequencing technique was utilized to analyze the fecal samples, with the objective of characterizing the composition and potential functional profile of the gut microbiota, as well as examining the correlations between the gut microbiota and disease-related clinical variables.

**Results:**

Compared with healthy controls, patients with adenomyosis showed a distinct gut microbial community composition. The adenomyosis group was significantly enriched in *Prevotella_9* and *Ligilactobacillus*, whereas the control group had an enrichment in *Anaerofustis*, *Akkermansia*, and *Anaerostipes*. PICRUSt2 results showed significant down-regulation of PWY0-1061 (superpathway of L-alanine biosynthesis), P184-PWY (protocatechuate degradation I (meta-cleavage pathway)), and DHGLUCONATE-PYR-CAT-PWY (glucose degradation (oxidative)) pathways in the ADS group. Correlation analysis further revealed that the relative abundances of *Akkermansia*, *Roseburia*, *Dialister*, and *Lachnoclostridium* were significantly associated with clinical features of the disease.

**Conclusion:**

A potential association exists between adenomyosis and the intestinal microbiome. Clinical characteristics of adenomyosis are related to the intestinal microbiota. These findings offer microbiota-derived evidence and new perspectives on the pathophysiology of adenomyosis.

## 1 Introduction

Adenomyosis (ADS) is characterized by the invasion of endometrial glands and stroma into the myometrium, accompanied by smooth muscle hypertrophy and hyperplasia (Zhai et al., 2020). Prevalence estimates range from 5 to 70%, with approximately 20% of reproductive-age individuals affected (Di Donato et al., 2014; Naftalin et al., 2012). Common symptoms include heavy menstrual bleeding (HMB), dysmenorrhea, pelvic pain, and subfertility (Bourdon et al., 2021a). ADS impairs both natural and assisted conception and is associated with elevated miscarriage rates (Horton et al., 2019). Non-invasive diagnostic tools and conservative treatments remain limited and do not offer a cure.

Over the years, the medical consensus on ADS has been that it is an estrogen-dependent disease characterized by growth and regression (Kitawaki, 2006). The prevailing theories include endometrium basalis invagination, tissue injury and repair, metaplasia of Müllerian duct remnants, adult stem cells differentiation, inflammatory stimulus, immunology, and epithelial-to-mesenchymal transition (Vannuccini et al., 2017; García-Solares et al., 2018; Bourdon et al., 2021b). However, existing pathogenic models cannot fully account for its complex disease mechanisms, suggesting that ADS pathogenesis may involve synergistic effects of additional factors. With the recent intensification of microbial research, growing evidence has linked the microbiota to ADS (Dantzler et al., 2025). Earlier studies primarily focused on the microbiota of the female reproductive tract (Kunaseth et al., 2022; Chao et al., 2021; Lin et al., 2023; Troha et al., 2026; Li J. et al., 2025; Li C. et al., 2025). More recent evidence indicates that the gut microbiota may represent a critical yet underexplored factor in disease progression (Chen et al., 2023; Valdés-Bango et al., 2024).

The gut microbiota is microbiota organism that can respond to growth processes and the environment (Shi et al., 2017). Gut microbiota can regulate digestion, metabolism, intestinal permeability, and immunological responses, and imbalances in the intestinal flora can cause an alteration in estrogen and a pro-inflammatory condition (Song et al., 2020; Ni et al., 2020; Gomaa, 2020). The gut microbiota can interact with estrogen through the enterohepatic circulation (Baker et al., 2017). Increased levels of circulating estrogen and intestinal bacteria producing *β*-glucuronidase are connected to endometriosis (Baker et al., 2017). Previous studies have discovered an association between endometriosis, pathological pregnancy, and ovarian cancer (Parpex et al., 2025; Wu et al., 2026; Tang et al., 2023; Lyu et al., 2023; Rosario et al., 2024). Moreover, the gut microbiota participates in immune-inflammatory processes through activation of pattern recognition receptors, particularly Toll-like receptors (TLRs). Pathogenic bacteria disrupt the intestinal mucosal barrier, enabling lipopolysaccharide (LPS) from Gram-negative bacteria to enter the circulatory system. This process activates the nuclear factor kappa-B (NF-κB) signaling pathway via Toll-like receptor 4 (TLR4), a member of the pattern recognition receptor family, thereby contributing to immune-inflammatory responses (Belkaid and Harrison, 2017; Salliss et al., 2021; Khan et al., 2013; Khan et al., 2009). The LPS/TLR4 signaling axis represents a critical pathway involved in inflammatory proliferation and invasive growth of ADS cells (Guo et al., 2016). Little is known about the connection between gut flora and ADS. A correlation between ADS and intestinal flora was initially found in animal experiments (Chen et al., 2023). Subsequent studies confirmed this association in patients with ADS, with a focus on analyzing the structural characteristics and biodiversity of the gut microbiota. However, part participants in this study received hormone therapy (Valdés-Bango et al., 2024). Therefore, evidence linking the gut microbiota to ADS is still lacking, even though ADS has been connected with alterations in the intestinal microbiome.

Previous studies have suggested that the clinical symptoms of ADS may be associated with abnormalities in steroid hormones, inflammation, cellular proliferation, and alterations in neuroangiogenesis (Vannuccini et al., 2017). However, the exact mechanisms remain unclear, and no effective targeted therapies are currently available. Recent research has indicated that gut microbiota may play a role in the development of ADS symptoms. The gut microbiota interacts bidirectionally with the central nervous system via the gut-brain axis (Canakis et al., 2020). Based on gut-brain axis interactions, gut microbiota regulates peripheral and central sensitization through various signaling molecules, thereby contributing to the pathogenesis of chronic pain (Guo et al., 2019). This mechanism may underlie the potential association with ADS-related dysmenorrhea. Furthermore, microbiota alterations in ADS may be associated with subfertility. Studies on infertility have demonstrated significant impacts on both the reproductive tract and gut microbiota (Marcos et al., 2024). The gut microbiota has been implicated in the development of endometriosis-associated infertility (Wang et al., 2024) However, the relationship between gut microbiota and ADS symptoms remains poorly investigated.

The primary objective of this study was to compare the structural characteristics of the gut microbiota between treatment-naïve female patients with ADS and healthy controls, to predict potential functional profiles of the microbial community, and to examine correlations between the gut microbiota and disease-related clinical variables.

## 2 Materials and methods

### 2.1 Study design and participants

**Figure 1:**
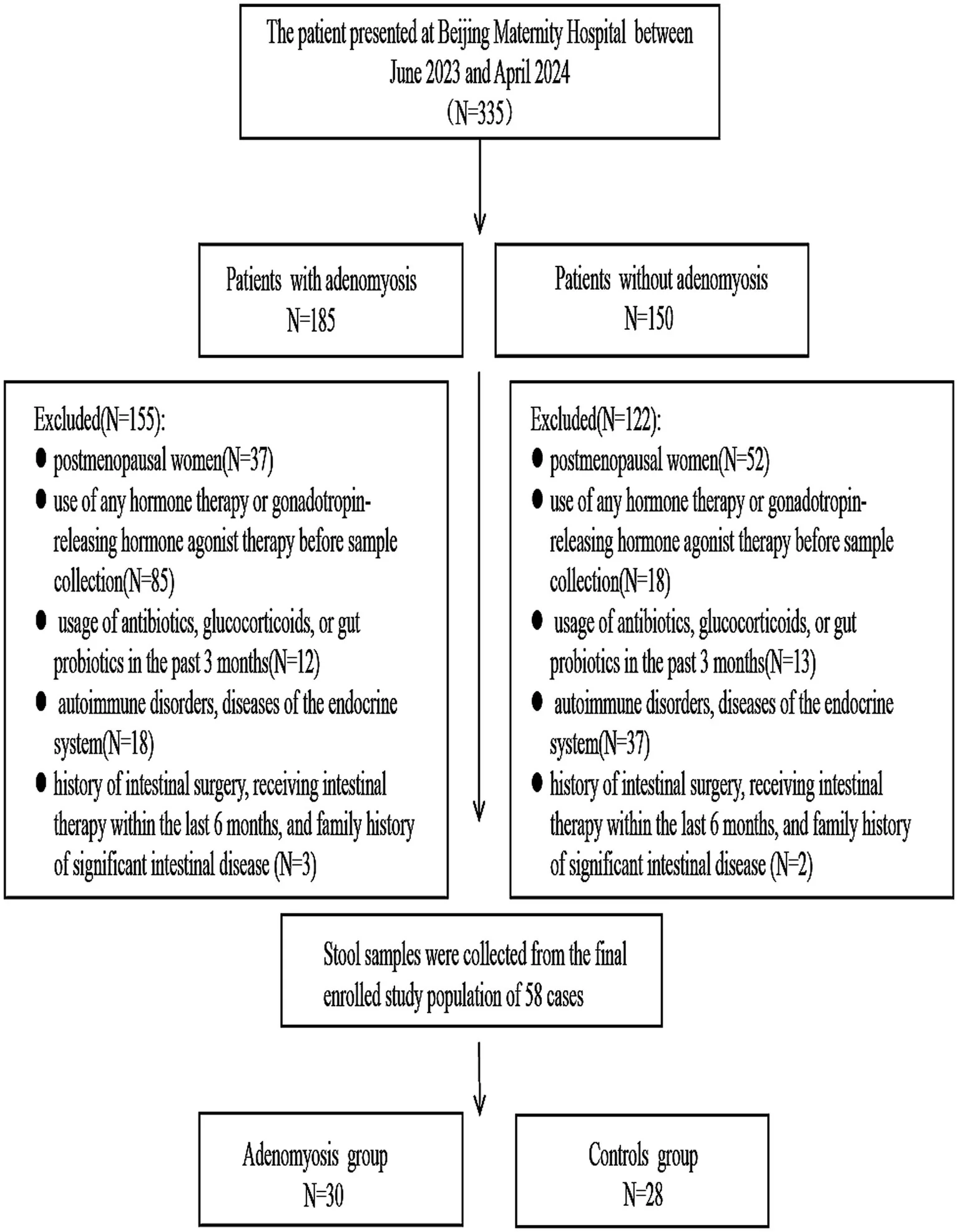
Flow chart.

This study enrolled women with untreated ADS and healthy controls from the Minimally Invasive Centre of Gynecology and Obstetrics, Beijing Maternity Hospital, Capital Medical University, between June 2023 and April 2024. All participants were premenopausal women aged 18–50 years, residing in northern China, and diagnosed with adenomyosis based on clinical symptoms, signs, and ultrasonographic findings. Ultrasonographic diagnosis followed the standard criteria defined by the Morphological Uterus Sonographic Assessment (MUSA) group (Van den Bosch et al., 2015; Harmsen et al., 2022) and was performed by experienced sonographers at the Beijing Obstetrics and Gynecology Hospital, affiliated with Capital Medical University. The control group consisted of healthy, asymptomatic women with no abnormalities detected in the uterus or bilateral adnexa during routine physical examination and ultrasonography. Exclusion criteria included:(1) postmenopausal women; (2) use of any hormone therapy or gonadotropin-releasing hormone agonist therapy before sample collection; (3) usage of antibiotics, glucocorticoids, or gut probiotics in the past 3 months; (4) autoimmune disorders, diseases of the endocrine system; and (5) history of intestinal surgery, receiving intestinal therapy within the last 6 months, and family history of significant intestinal disease. The enrollment flow chart is shown in Figure 1, and 58 subjects were finally included. The hospital ethics committee approved the study (No. 2019-KY-105-01). Detailed clinical data were gathered from the electronic medical record database and handwritten documents.

### 2.2 Specimen collection

Subjects collected fresh fecal samples in the hospital. Before sample collection, patients voided urine and avoided menstruation, then collected the mid-portion of feces. Approximately 1 g aliquots were weighed and placed in separate containers. Samples were transported on dry ice within 30 min of collection and stored at −80 °C until DNA extraction.

### 2.3 DNA extraction, PCR amplification, and sequencing

Microbial DNA was extracted from fecal specimens using the TIANamp Soil DNA Kit (TIANGEN, China) according to the manufacturer’s instructions. The V3-V4 regions of the 16S rRNA gene were amplified with primers 341F (5’-CCTAYGGGRBGCASCAG-3′) and 806R (5’-GGACTACNNGGGTATCTAAT-3′). The PCR reaction was operated by the following procedure: 1 min of 98 °C denaturation, 30 cycles of 10 s of 98 °C denaturation, 30 s of annealing at 50 °C, 30 s of elongation at 72 °C, and 5 min at 72 °C. Forward Primer and reverse primer of 0.2 μM, 15 μL of Phusion® High-Fidelity PCR Master Mix (New England Biolabs, Ipswich), and about 10 ng of template DNA were used in all PCR reactions. PCR amplified products were quantified by the Agilent 5,400 Fragment Analyzer System (Agilent Technologies, USA). PCR amplification products were recovered using the Universal DNA product purification and recovery kit (TIANGEN, CHINA). The amplified PCR products were purified using XP Bead Purification (Beckman Coulte, USA). The purified PCR products were used for library preparation using the NEBNext Ultra II DNA Library Prep Kit (Illumina, USA). The Agilent 5,400 Fragment Analyzer System (Agilent Technologies, USA) and QuantStudio™ 12 K Flex qPCR (Thermo Fisher, USA) were used in conjunction for library quality control. The sequencing work was completed on the Illumina NovaSeq6000 sequencing platform.

### 2.4 Bioinformatics analysis

Metadata analysis was conducted using QIIME2 software (version 2022.2). The raw sequencing data were spliced by Fast Length Adjustment of Short reads (FLASH) (Magoč and Salzberg, 2011), matched with reverse primer sequences, and cut out the remaining sequences by Cutadapt and fastp software (Version 0.23.1) for quality control. Removal of chimeras contrasted with the Silva database.^1^ The Amplicon Sequence Variant (ASV) was obtained by noise reduction using the divisive amplicon denoising algorithm 2 (DADA2) module of the QIIME2 software. The annotation of species was accomplished through the Silva138.1 database. Gut microbiota diversity detection through the QIIME2 platform for Observed species, Chao1 index, Shannon index, and Simpson index in Alpha diversity. The higher the number of Observed species, the higher the species richness. A greater Chao1 index indicates a higher gut microbiota abundance. The greater the Shannon index, the higher the community diversity and the more evenly distributed the species. The Simpson index represents the diversity and evenness of species distribution in the community. The closer the Simpson index is to 1, the higher the community diversity and the more uniform the species distribution. Βeta diversity was analyzed by the Unifrac distance algorithm and further analyzed using Principal Co-ordinates Analysis (PCoA) and graphically presented using the weighted UniFrac algorithm. The higher the structural similarity of the sample colonies, the more the distances tend to cluster together in the PCoA. Gut flora biomarker screening was performed using the Linear discriminative analysis effect size (LEfSe) algorithm to estimate the effect of the abundance of each species on the differential effect, and Linear discriminant analysis (LDA) scores were calculated for these genera to assess their influence, with the LDA threshold set at 2. Species with an LDA value >2 were identified as potential gut microbiota markers. Predicting the enrichment of functional pathways in microbiota applied the Phylogenetic Investigation of Communities by Reconstruction of Unobserved States version 2.0 (PICRUSt2.0) software (version 2.3.0) by annotation and comparison with genes in the Integrated Microbial Genomes (IMG) database. Spearman correlation tests were performed in combination with environmental factors.

Metadata analysis was conducted using QIIME2 software (version 2022.2). The raw sequencing data were spliced by Fast Length Adjustment of Short reads (FLASH) (Magoč and Salzberg, 2011), matched with reverse primer sequences, and cut out the remaining sequences by Cutadapt and fastp software (Version 0.23.1) for quality control. Removal of chimeras contrasted with the Silva database (see Footnote 1). The Amplicon Sequence Variant (ASV) was obtained by noise reduction using the divisive amplicon denoising algorithm 2 (DADA2) module of the QIIME2 software. The annotation of species was accomplished through the Silva138.1 database. Sample data were rarefied to the minimum sequencing depth across all samples. ASV absolute abundance was normalized based on the sequencing depth of the sample with the fewest sequences. A rarefaction curve was constructed to evaluate whether sequencing depth was sufficient. Gut microbiota diversity detection through the QIIME2 platform for Observed species, Chao1 index, Shannon index, and Simpson index in Alpha diversity. The higher the number of Observed species, the higher the species richness. A greater Chao1 index indicates a higher gut microbiota abundance. The greater the Shannon index, the higher the community diversity and the more evenly distributed the species. The Simpson index represents the diversity and evenness of species distribution in the community. The closer the Simpson index is to 1, the higher the community diversity and the more uniform the species distribution. Βeta diversity was analyzed by the Unifrac distance algorithm and further analyzed using Principal Co-ordinates Analysis (PCoA) and graphically presented using the weighted UniFrac algorithm. The higher the structural similarity of the sample colonies, the more the distances tend to cluster together in the PCoA. Biomarker screening for gut microbiota was performed using the Linear discriminative analysis effect size (LEfSe) algorithm to estimate the contribution of each species’ abundance to differential effects. Linear discriminant analysis (LDA) scores were calculated to evaluate these effects, with an LDA threshold set at 2. Species with an LDA value greater than 2 were considered potential gut microbial markers. Functional pathway enrichment within the microbial community was predicted using Phylogenetic Investigation of Communities by Reconstruction of Unobserved States version 2.0 (PICRUSt2.0) software (version 2.3.0), following gene annotation and comparison against the Integrated Microbial Genomes (IMG) database. Spearman correlation tests were performed in combination with environmental factors.

### 2.5 Statistical analysis

R package (version 4.0.3) and SPSS version 24.0 (IBM, USA) were utilized for statistical analysis. Alpha diversity index intergroup differences were examined by the Wilcox rank-sum test. Βeta diversity based on weighted Unifrac metrics for principal coordinates analysis (PCoA). Anosim analysis was employed to judge differences in community structure. The statistical biomarkers were found using Linear discriminant effect size (LEfSe) (Segata et al., 2011). Using Spearman’s rank correlation, the relationship between clinical characteristics and species was examined. According to the data type, clinical data were compared using the t-test, Mann–Whitney U-test, and Chi-square test. *p* < 0.05 signifies statistically significant variations.

## 3 Results

### 3.1 Baseline characteristics

A total of 58 subjects were included in this study, including 30 patients with ADS in the study group and 28 healthy people in the control group. The age of the patients in the ADS group was 41.8 ± 5.5 years and 38.9 ± 6.5 years in the control group; the difference was not statistically significant (*p* > 0.05). Body mass index (BMI) was 22.2 ± 2.9 kg/m^2^ in the ADS group and 22.8 ± 2.6 kg/m^2^ in the control group; the difference was not statistically significant (*p* > 0.05). The number of gravida was 2 (1–3) times in the ADS group and 2 (0–2) times in the control group, a statistically significant difference (*p* < 0.05). Patients in the ADS group had a dysmenorrhea score of 7 (3–8), and the control group had a dysmenorrhea score of 0 (0–0), a statistically significant difference (*p* < 0.001). Increased menstrual flow in the ADS group compared to the control group (50% vs. 0%, *p* < 0.001). The uterine volume of the patients in the ADS group was 128.5 (83.4–230.6) cm^3^, and the uterine volume of the control group was 53.6 (39.1–62.8) cm^3^, which was statistically significant (*p* < 0.001). Regarding parity, menstrual cycle, and menses duration, the difference is not statistically significant (Table 1).

**Table 1:** Baseline statistics of the ADS group and the control.

Characteristics	ADS (*n* = 30)	Control (*n* = 28)	*p* value
Age (years)	41.8 ± 5.5	38.9 ± 6.5	0.066
BMI (kg/m^2^)	22.2 ± 2.9	22.8 ± 2.6	0.414
Gravida (times)	2 (1–3)	2 (0–2)	0.033
Parity (times)	1 (1–1)	1 (0–1)	0.283
Menses duration	6 (5–7)	5.5 (4.3–6.8)	0.123
Menstrual cycle	28 (25–30)	28 (25.3–30)	0.860
VAS score	7 (3–8)	0 (0–0)	<0.001
HMB (*n*, %)	15 (50)	0 (0)	<0.001
Uterine volume (cm^3^)	128.5 (83.4–230.6)	53.6 (39.1–62.8)	<0.001

Data are displayed as the mean ± SD, *M*(P25, P75), or *n* (%).

BMI, body mass index; VAS, visual analog scale; HMB, heavy menstrual bleeding.

### 3.2 Characterization of 16S rRNA sequencing data

From 58 samples, 6,841,815 raw reads (117,962.328 reads per sample) and 5,690,242 high-quality clean reads (98,107.621 reads per sample) were finally acquired (Supplementary Table S1). The alpha rarefaction curve reached a plateau as the sequencing depth increased. Good’s coverage approach is 1.0, indicating that the sequencing depth is sufficient for subsequent sequencing requirements (Supplementary Figure S1).

### 3.3 Structural species composition and abundance

**Figure 2:**
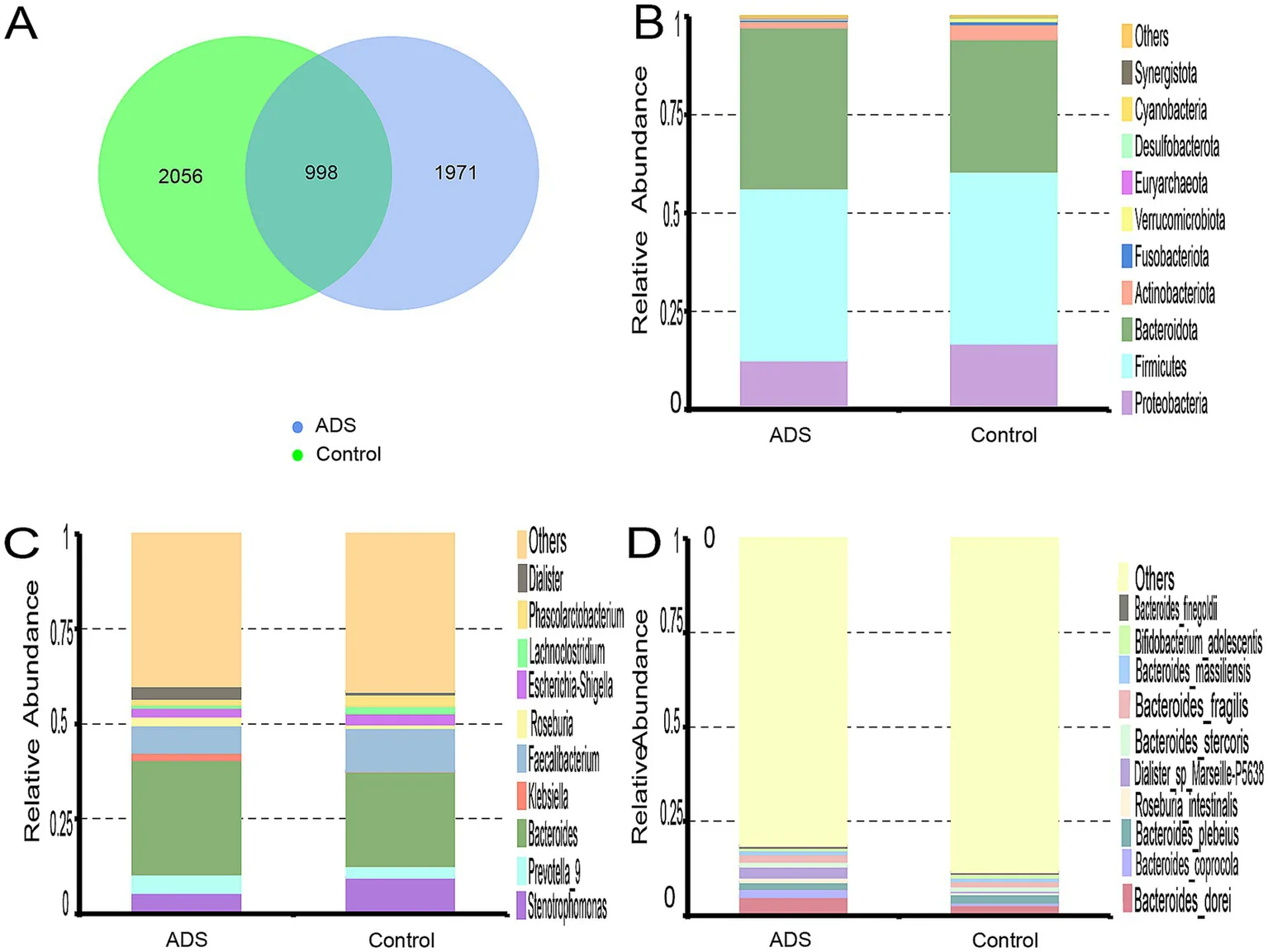
Shared and unique microbial features and taxonomic composition of the gut microbiota in patients with ADS and HC. **(A)** Venn diagram showing the overlap of ASV between the ADS group (*n* = 30) and the healthy control group (*n* = 28). A total of 998 ASVs were shared between the two groups, whereas 1971 and 2056 unique ASV were identified in the ADS and HC groups, respectively. **(B–D)** Bar charts depicting the relative abundance of the top 10 most abundant taxa at the **(B)** phylum, **(C)** genus, and **(D)** species levels. Taxa not ranked among the top 10 were grouped into the “Others” category. ADS, adenomyosis; HC, healthy control; ASV, amplicon sequence variant.

The Venn diagram revealed 998 ASV in the two groups, with 1971 and 2056 unique ASV in each of the ADS and control groups (Figure 2A). In terms of phylum, the four primary intestinal flora in both groups were *Firmicutes*, *Bacteroidota*, *Proteobacteria*, and *Actinobacteriota*, with *Firmicutes* having the highest proportion, over 40% in both groups. *Bacteroidota*, *Proteobacteria*, and *Firmicutes* made up over 90% of the entire flora (Figure 2B). In terms of the genus, the predominant flora in ADS was *Bacteroides* (30.0%), *Faecalibacterium* (7.2%), *Stenotrophomonas* (5.5%), *Prevotella_9* (4.9%), and *Dialister* (3.3%). *Bacteroides* (24.8%), *Faecalibacterium* (11.4%), *Stenotrophomonas* (9.5%), *Phascolarctobacterium* (3.2%), and *Prevotella_9* (3.2%) were the dominant microorganisms in the healthy control people (Figure 2C). In terms of species, the predominant flora in ADS were *Bacteroides_dorei* (5.0%), *Dialister_sp_Marseille-P5638* (2.9%), and *Bacteroides_coprocola* (2.1%). *Bacteroides_dorei* (2.9%), *Bacteroides_plebeius* (2.2%), and *Bacteroides_fragilis* (1.3%) were the most common microorganisms in the healthy population (Figure 2D).

### 3.4 Diversity of gut microbiota

**Figure 3:**
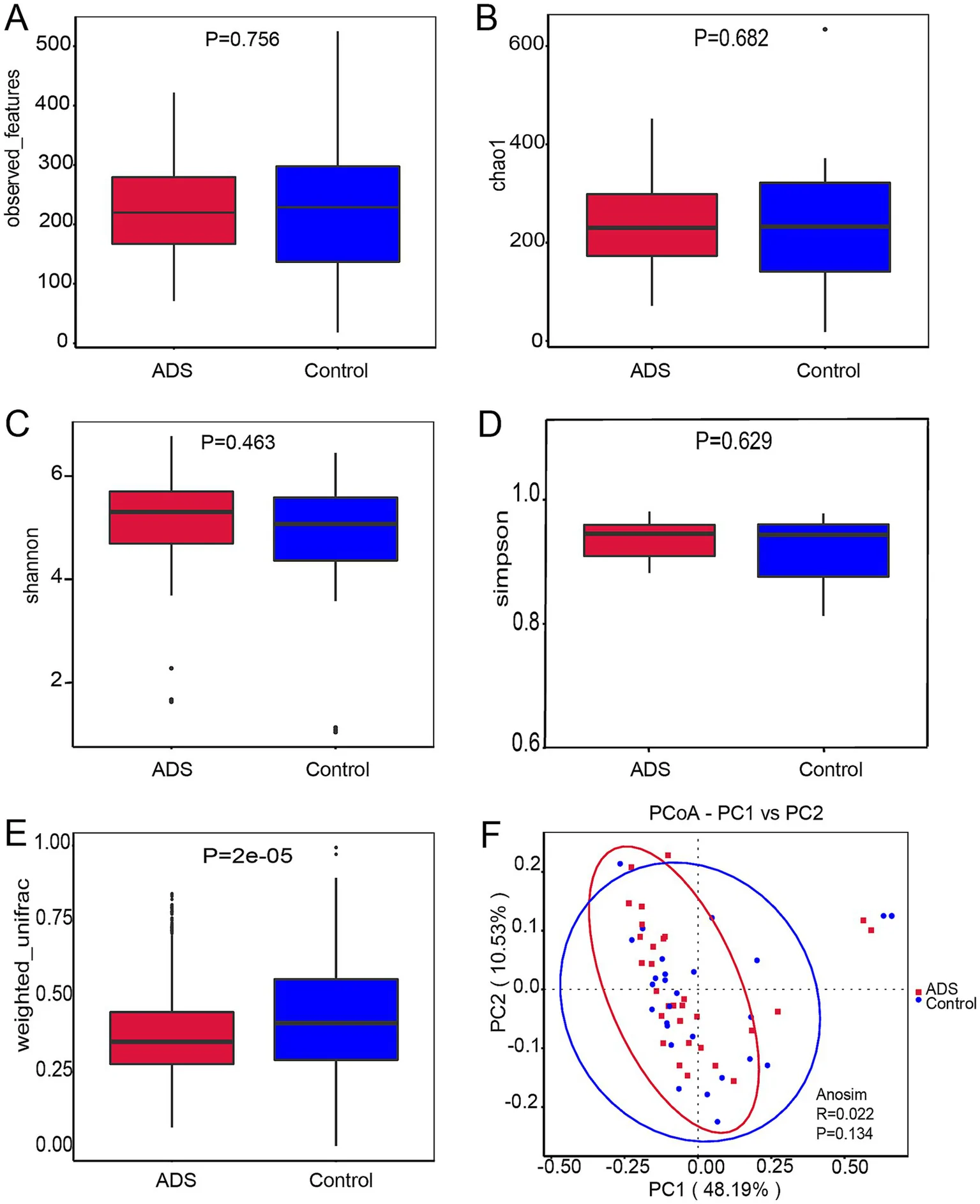
Gut microbiota alpha diversity and beta diversity in ADS and control groups. **(A)** Number of observed species. Observed species were 2,969 in the ADS group and 3,054 in the control group (*p* = 0.756). **(B)** The Chao1 index was 3196.268 in the ADS group and 3301.163 in the control group (*p* = 0.682). **(C)** Shannon index was 7.755 in the ADS group and 7.479 in the control group (*p* = 0.463). **(D)** Simpson index was 0.988 in the ADS group and 0.982 in the control group (*p* = 0.629). **(E)** The beta diversity analysis is based on weighted UniFrac distance (*p* < 0.0001). **(F)** Beta diversity was examined using Principal Coordinates Analysis (PCoA). In the PCoA plot, each point represents an individual sample (red = ADS, blue = HC). Ellipses (95% confidence intervals) indicate the distribution of group clustering. PC1 explained 48.19% of the variation, and PC2 accounted for 10.53% of the variation. ANOSIM showed no significant grouping effect (*R* = 0.022, *p* = 0.134).

The results of gut microbiota Alpha diversity between the two groups showed that Observed species were 2,969 in the ADS group and 3,054 in the control group; the difference was not statistically significant (*p* = 0.756). The Chao1 index was 3196.268 in the ADS group and 3301.163 in the control group; the difference was not statistically significant (*p* = 0.682). The ADS group exhibited a Shannon index of 7.755 compared to 7.479 in controls, while Simpson indices were 0.988 and 0.982, respectively. These results indicate that the ADS group had a higher diversity of intestinal communities and a more homogeneous species distribution. However, none of the differences were statistically significant (*p* > 0.05) (Figures 3A–D) (Supplementary Table S2). βeta diversity showed significant differences (*p* < 0.0001) (Figure 3E). PCoA analyses were used to evaluate βeta diversity through the weighted UniFrac, and two groups generated different clusters, with PC1 explaining 48.19% of the variance, and PC2 explaining 10.53% of the variance. The anosim analysis suggests that the grouping makes sense (*R* = 0.022) (Figure 3F).

### 3.5 Gut microbiota biomarker analysis

**Figure 4:**
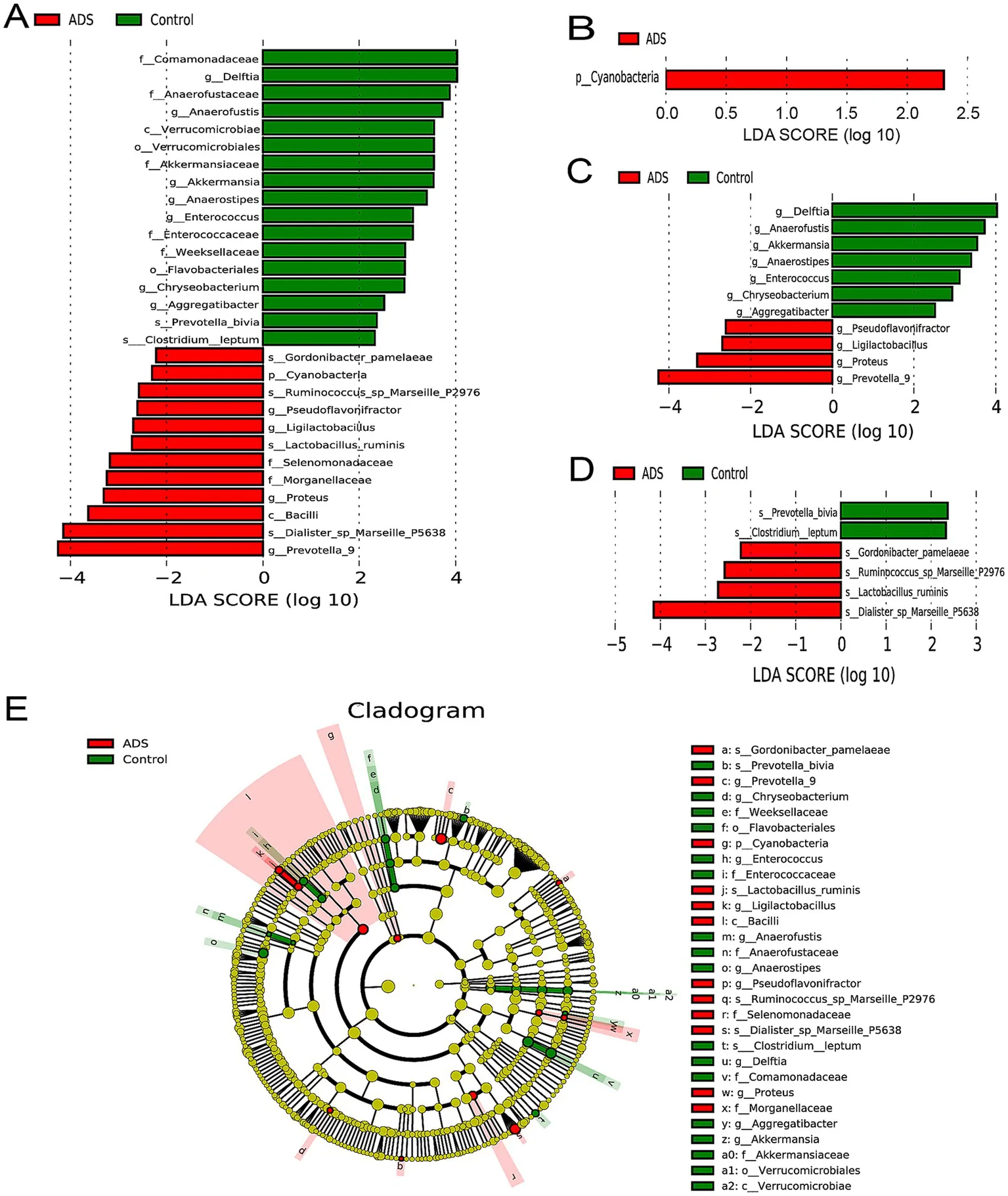
Intergroup differences among species in ADS and control groups. Histograms of **(A–D)** LDA scores are shown. **(A)** LEfSe analysis was performed at the full species level, with species having LDA scores greater than 2 considered potential gut microbiota markers. Only species with *p* < 0.05 are displayed. Red bars indicate taxa significantly enriched in the ADS group, whereas green bars denote those enriched in the HC group. **(B–D)** Abundance differences at specific taxonomic levels are presented, with significantly enriched results shown at the phylum, genus, and species levels, respectively. **(B)** C*yanobacteria* are significantly enriched in the ADS group at the phylum level. **(C)** *Prevotella_9*, *Proteus*, *Ligilactobacillus*, and *Pseudoflavonifractor* were notably enriched in ADS at the genus level (*p* < 0.05), whereas the control group featured significantly more abundances of *Delftia*, *Anaerofustis*, *Akkermansia*, *Anaerostipes*, *Enterococcus*, *Chryseobacterium*, and *Aggregatibacter* (*p* < 0.05). **(D)** *Dialister_sp_Marseille_P5638*, *Lactobacillus_ruminis*, *Ruminococcus_sp_Marseille_P2976*, and *Gordonibacter_pamelaeae* (*p* < 0.05) are significantly enriched in the ADS group at the species level. **(E)** A cladogram of the taxonomic hierarchy was generated based on the LEfSe analysis results. Red nodes represent significantly higher abundance in the ADS group, green nodes indicate significantly higher abundance in the HC group, and yellow nodes denote species with no significant differences between the groups.

LEfSe analysis untangled potential bacterial biomarkers, and the degree of influence of differential flora on classification was assessed by LDA score (LDA > 2, *p* < 0.05). Bar charts and cladograms illustrated the distribution of characteristic genera across taxonomic levels. The analysis detected significant abundance variations in 29 species—12 in ADS and 17 in controls. In terms of phylum, *Cyanobacteria* are significantly enriched in the ADS group. *Prevotella_9*, *Proteus*, *Ligilactobacillus*, and *Pseudoflavonifractor* were notably enriched in ADS at the genus level (*p* < 0.05), whereas the control group featured significantly more abundances of *Delftia*, *Anaerofustis*, *Akkermansia*, *Anaerostipes*, *Enterococcus*, *Chryseobacterium*, and *Aggregatibacter* (*p* < 0.05). In terms of species, the ADS exhibited a high enrichment of species such as *Dialister_sp_Marseille_P5638*, *Lactobacillus_ruminis*, *Ruminococcus_sp_Marseille_P2976*, and *Gordonibacter_pamelaeae* (*p* < 0.05). On the other hand, the control group had greater levels of *Prevotella_bivia* and *Clostridium__leptum* (*p* < 0.05) (Figure 4).

### 3.6 Microbial community functionality prediction

**Figure 5:**
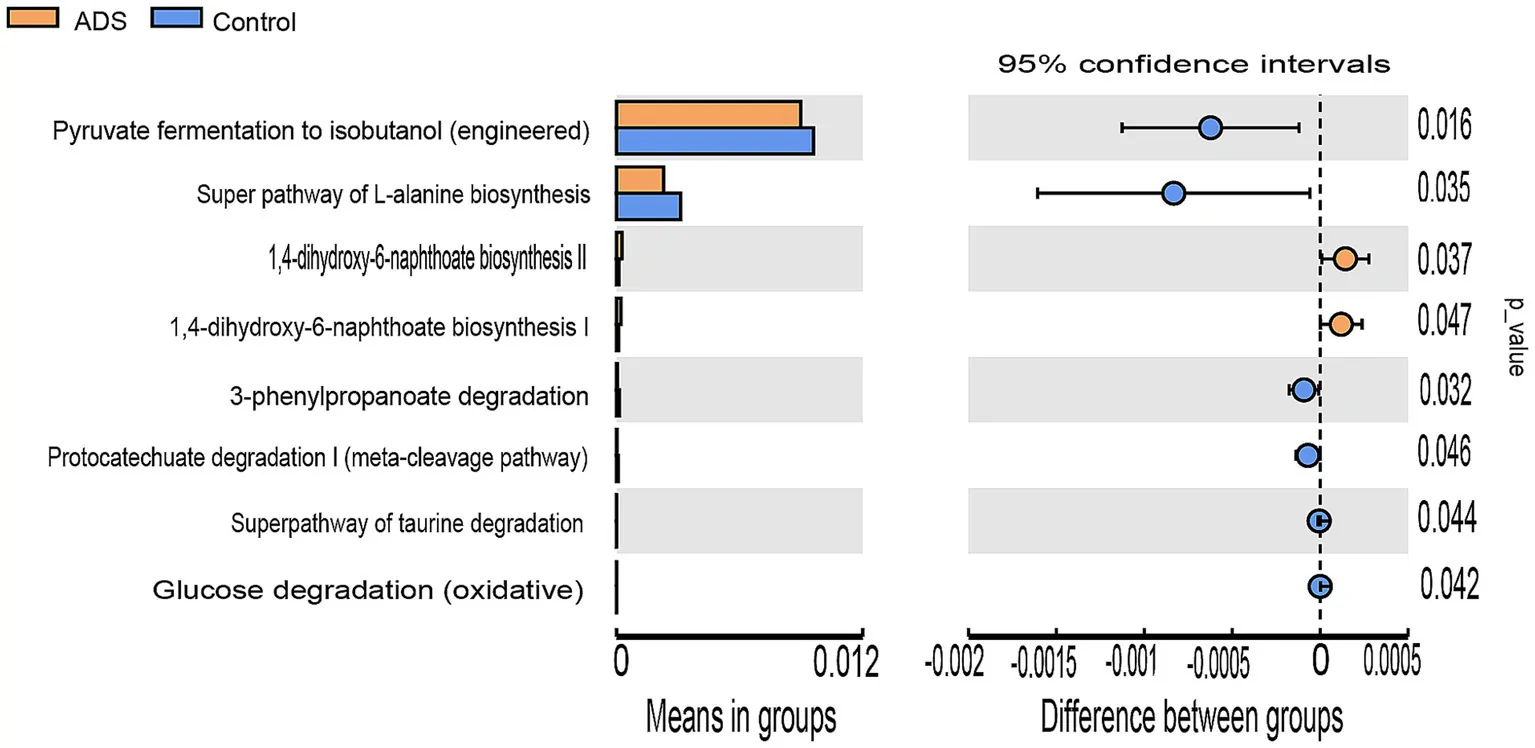
Functional pathway of the intestinal microbiota in the ADS and control groups. PWY-7371 (1,4-dihydroxy-6-naphthoate biosynthesis II) and PWY-7374 (1,4-dihydroxy-6-naphthoate biosynthesis I) were upregulated in ADS (*p* < 0.05). PWY-7111 (pyruvate fermentation to isobutanol (engineered)), PWY0-1061 (super pathway of L-alanine biosynthesis), P281-PWY (3-phenylpropanoate degradation), P184-PWY (protocatechuate degradation I (meta-cleavage pathway)), PWY-1541 (superpathway of taurine degradation), and DHGLUCONATE-PYR-CAT-PWY (glucose degradation (oxidative)) pathways were downregulated in ADS (*p* < 0.05). Control is shown in blue and the ADS group in orange.

PICRUSt2 predicted functional profiles of intestinal microbiota. The intestinal flora of the ADS group is considerably enriched in the metabolic pathway relating to PWY-7371 (1,4-dihydroxy-6-naphthoate biosynthesis II) and PWY-7374 (1,4-dihydroxy-6-naphthoate biosynthesis I). Compared to the control group, the PWY-7111 (pyruvate fermentation to isobutanol (engineered)), PWY0-1061 (super pathway of L-alanine biosynthesis), P281-PWY (3-phenylpropanoate degradation), P184-PWY (protocatechuate degradation I (meta-cleavage pathway)), PWY-1541 (superpathway of taurine degradation), and DHGLUCONATE-PYR-CAT-PWY (glucose degradation (oxidative)) pathways were significantly down-regulated in ADS (Figure 5).

### 3.7 Correlation between intestinal microbiota and ADS clinical characteristics

**Figure 6:**
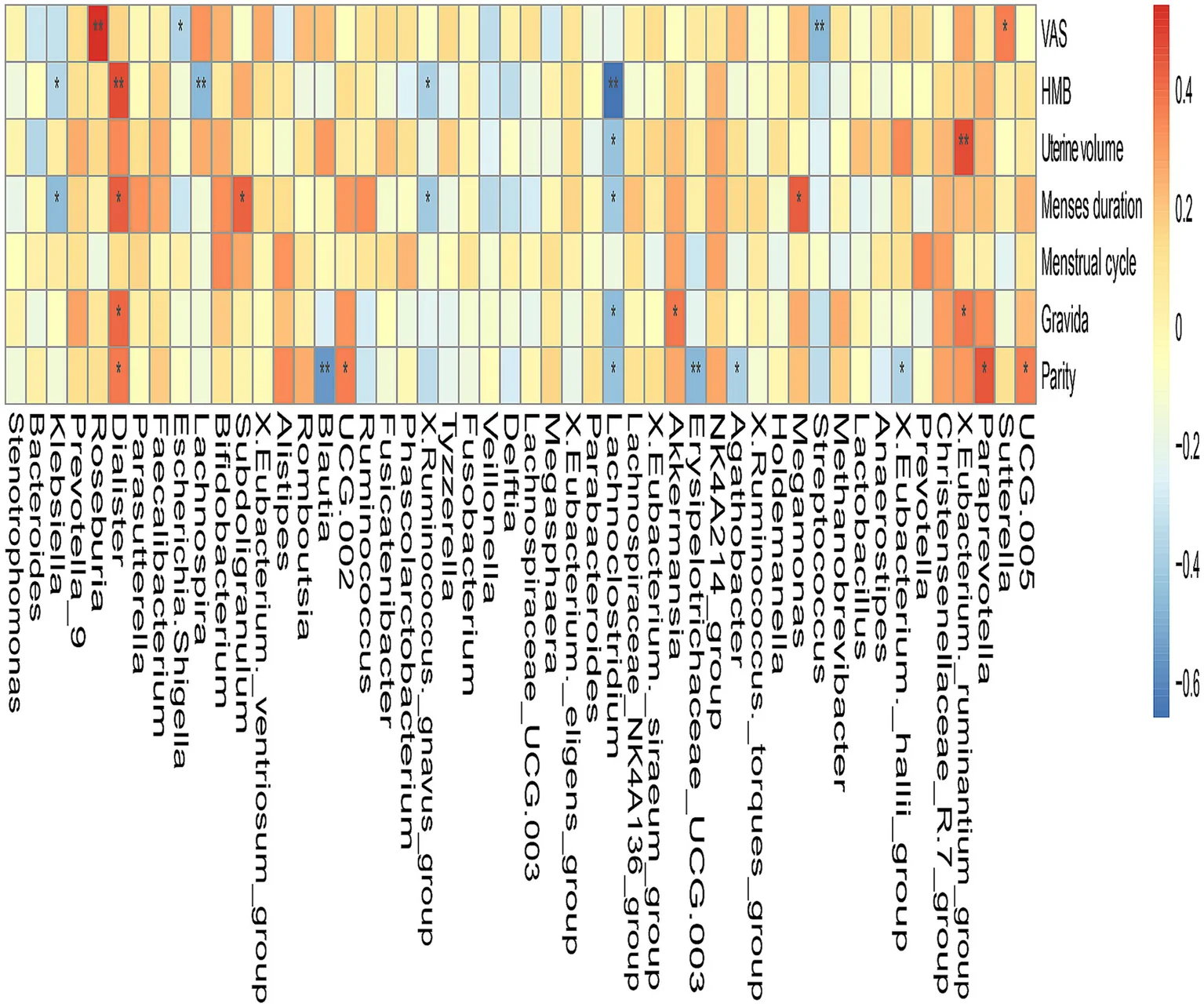
Heatmap of correlation analysis between gut microbiota and clinical features. Each row of the graph represents an environmental factor, and each column represents a differential species (genus level). (+) means that the environmental factor correlates with the corresponding species, red represents a positive correlation, and blue a negative correlation. *p < 0.05; **p < 0.01.

We conducted Spearman’s correlation analyses with clinical features of VAS, HMB, gravida, parity, menstrual cycle, menses duration, and uterine volume to evaluate the interaction among clinical features and gut microbiota. In terms of genus, we screened the top 50 species based on abundance. The results showed that VAS, HMB, gravida, parity, menses duration, and uterine volume were associated with a variety of genera in ADS and were statistically significant (*p* < 0.05). *Akkermansia* demonstrated a significant positive correlation with gravidity (*r* = 0.382, *p* = 0.037), while *Roseburia* showed a marked positive correlation with dysmenorrhea severity (*r* = 0.542, *p* = 0.002). Notably, *Dialister* and *Lachnoclostridium* are associated with menstrual conditions and fertility status. *Dialister* demonstrated positive correlations with HMB (*r* = 0.472, *p* = 0.008), menses duration (*r* = 0.442, *p* = 0.014), gravida (*r* = 0.413, *p* = 0.023), and parity (*r* = 0.385, *p* = 0.036). Conversely, *Lachnoclostridium* showed negative correlations with HMB (*r* = −0.659, *p* = 0.00008), menses duration (*r* = −0.417, *p* = 0.022), gravida (*r* = −0.463, *p* = 0.01), parity (*r* = −0.434, *p* = 0.017), and uterine volume (*r* = −0.438, *p* = 0.016) (Figure 6).

## 4 Discussion

ADS is a common benign, and refractory gynecologic disease. In recent years, a variety of pathogenic hypotheses have been proposed. Local relative hyperestrogenism may be involved in the initial stage of tissue injury and repair, as well as in the invagination of the endometrial basal layer (García-Solares et al., 2018). Furthermore, inflammatory states and immune alterations play pivotal roles in key pathogenic processes, including tissue damage and repair, proliferation, invasion, and ectopic lesion survival (Vannuccini et al., 2017; Bourdon et al., 2021b). Numerous pathogenetic theories still fail to fully explain the occurrence of the disease, and with diverse clinical manifestations and unsatisfactory conservative treatments, the disease remains a great challenge. Dysbiosis of the gut flora can participate in estrogen-related disease processes (Baker et al., 2017). The balance of commensal and pathogenic intestinal bacteria is essential for immune regulation. Commensal flora promotes the refinement of the mucosal immune system, whereas pathogenic microbiota induce disease by interfering with immune homeostasis (Shi et al., 2017). Therefore, based on similar pathogenesis, the research used 16 s rRNA gene sequencing to investigate the gut microbiota features and relationship with clinical variables of women with ADS and healthy controls.

The 16S rRNA amplicon sequencing revealed no significant difference in intestinal microbiota Alpha diversity between ADS and controls, βeta diversity was significantly different. Earlier investigations have identified changes in alpha diversity and βeta diversity of intestinal flora in populations with ADS (Valdés-Bango et al., 2024). However, some patients in those studies had received hormone therapy. In contrast, no diversity differences were found in the ADS mouse gut flora study (Chen et al., 2023). In terms of species composition, the level of *Bacteroidota* increased in the ADS group. *Bacteroides* is the main bacterial group causing the LPS/TLR4 inflammatory pathway in the intestinal flora (Li S. et al., 2021).

Further LEfSe results showed enrichment of *Cyanobacteria* in the ADS group. The outer membrane of *Cyanobacteria* contains endotoxin LPS, which has been demonstrated to control innate immune responses by means of TLR4 signaling (Durai et al., 2015). Hyperactivation of TLR4 results in autoimmune and inflammatory illnesses (Kondo et al., 2012).

Our study found an increase in another flora associated with inflammation in the ADS group. *Prevotella*, *Bacteroidetes* phylum, is an anaerobic Gram-negative bacterium. *Prevotella* is an inflammation-associated microbiota linked to a variety of illnesses, including periodontitis, peritonitis, ulcerative colitis, and bacterial vaginosis (Larsen, 2017). *Prevotella* was found to be upregulated in the ADS group in our study. Elevated *Prevotella* abundance was also found in our previous research on endometrial microbiota in ADS and served as a potential biomarker for ADS (Lin et al., 2023). Under dysbiotic conditions, *Prevotella* shifts from commensalism to pathogenicity, modulating host immune responses and upregulating virulence factors such as LPS, hemolysins, adhesins, proteases, and quorum-sensing molecules (Sharma et al., 2022). TLR4 was observed to exist in ADS in endometrial stromal cells, and the expression of TLR4 signaling pathway-related molecules, TLR4, MD2, MyD88, and NF-κB, was increased in LPS-treated endometrial stromal cells. Furthermore, LPS was found to increase the expression of proliferative and invasive factors and stimulate ADS stromal cells (Guo et al., 2016). Immune modulation and inflammatory imbalance in ADS may be associated with impaired endometrial receptivity (Horton et al., 2019). Further research on *Prevotella* can explore the inflammatory alterations and subfertility associated with ADS.

Estrogens and intestinal microbiota interact in a bidirectional manner. The “Estrobolome,” a gene repertoire for metabolizing estrogens in the intestine, is perceived as a regulator of the intestinal microbiota (Plottel and Blaser, 2011). Estradiol interacts with microorganisms and the host. Numerous biological functions, including metabolism, inflammation, cell proliferation, apoptosis, and differentiation, are significantly influenced by it (Edwards, 2005). In our study, we found an upregulated of *Ligilactobacillus* in the ADS group. Similarly, *Lactobacillus* was upregulated in mice with ADS’s intestinal microbiota (Chen et al., 2023). Research investigating the possibility of *Ligilactobacillus salivarius* strains participating in estrogen metabolism found that there is genetic potential for *Lactobacillus salivarius* strains to break down estrogens and/or participate in conjugation/deconjugation mechanisms (Aragón et al., 2024). Elevated local estrogen levels and increased production of aromatase and 17β-hydroxy cholesterol dehydrogenase of ADS promote the development of ectopic endometrium, which ultimately contributes to the formation of ADS (Maia et al., 2009). *Ligilactobacillus salivarius* strains are prone to bind to 17β-estradiol and carry two dehydrogenases encoding genes that function similarly to 17β-hydroxysteroid dehydrogenase (Aragón et al., 2024). Future work could continue to explore the estrogenic regulatory role of *Lactobacillus* with ADS.

The study revealed significantly lower enrichment of *Anaerofustis*, *Akkermansia*, and *Anaerostipes* in the ADS group. *Akkermansia*, an anaerobic, gram-negative bacterium from the *Verrucomicrobia* division (Derrien et al., 2010), modulates inflammatory cytokine levels and enhances intestinal barrier function through multiple signaling pathways (Li et al., 2023). Diminished *Akkermansia* abundance correlates with thinning of the intestinal mucus layer and compromised barrier integrity (Reunanen et al., 2015). Previous work has documented reduced *Akkermansia* levels in diabetes (Li et al., 2023), while a dehydroepiandrosterone-induced polycystic ovary syndrome mouse model also exhibited decreased intestinal *Akkermansia.* In contrast, metformin and disulfiram reduce IFN-*γ* levels, suppress macrophage pyroptosis, and enhance intestinal *Akkermansia* abundance (Huang et al., 2022).

Notably, the ADS group had a lower abundance of *Anaerofustis*, *Akkermansia*, and *Anaerostipes*, which are connected to the generation of butyrate (Finegold et al., 2004; Li Z. et al., 2021; Van-Wehle and Vital, 2024). An important source of energy for colon cells, butyric acid is a short-chain fatty acid that can also increase the production of tight junction proteins in the colon epithelium. It has anti-inflammatory, immune system, and metabolic pathway-influencing effects (Nicholson et al., 2012; Vital et al., 2017). Thus, lower butyrate concentrations may promote instability of the intestinal barrier (Plöger et al., 2012).

PICRUSt2 results identified that the ADS group in PWY0-1061 (superpathway of L-alanine biosynthesis), P184-PWY (protocatechuate degradation I (meta-cleavage pathway)), and DHGLUCONATE-PYR-CAT-PWY (glucose degradation (oxidative)) pathway were down-regulated. Previous studies have shown that Protocatechuic acid (PCA) can be cleaved via the meta-cleavage pathway and enter the TCA cycle (Tsagogiannis et al., 2021). Under normal gut microbiota conditions, butyrate can be transported via intestinal microbes into the mitochondria of colonic cells, participate in the Tricarboxylic Acid (TCA)cycle, and provide energy (Harty, 2013). Similarly, the DHGLUCONATE-PYR-CAT-PWY (glucose degradation (oxidative)) is a core component of carbohydrate catabolism; glucose provides energy for the small intestine. When butyrate levels decrease and colonic cells experience energy deficiency, the redox state of colonic cells changes, leading to reduced oxidative phosphorylation and increased oxidative stress (Harty, 2013). Alanine is a part of peptidoglycan that forms the cell wall (Reitzer, 2004). Bacterial peptidoglycan (PG) is one of the main types of pathogen-associated molecular patterns (Schleifer and Kandler, 1972). Nucleotide-binding oligomerization domain 2 (NOD2), an intracellular Pattern Recognition Receptor (PRR), recognizes muramyl dipeptide, a structural subunit of the bacterial cell-wall peptidoglycan. Subsequently, NOD2 interacts with receptor-interacting protein 2 (RIP2) to generate the NOD2-RIP2 complex. Finally, it activates NF-κB and MAPK-related inflammatory pathways and proinflammatory cytokine release (Jeong et al., 2014; Zhou et al., 2021). NOD2, located in the intestinal epithelium, plays a role in intestinal antimicrobial defense and the regulation of the intestinal microbiota (Duerr et al., 2011). Recent research identifies NOD2 as a hepatic mediator that senses intestinal dysbiosis and responds to gut-derived bacterial PAMP. NOD2 activates the MAPK, NF-κB, and STAT3 pathways through RIP2-dependent signaling, promoting hepatic inflammation (Zhou et al., 2021). Therefore, based on the above predictions regarding bacterial metabolic functions, alterations in microbial carbohydrate metabolism, energy metabolism, and cell wall biosynthesis may contribute to changes in the intestinal microenvironment of ADS patients.

Additionally, Spearman’s correlation analysis revealed correlations between the intestinal microorganisms *Roseburia*, *Akkermansia*, *Dialister* and *Lachnoclostridium* and the clinical features of ADS. The connection between microorganisms and clinical characteristics is noteworthy in ADS, a disease with multiple clinical manifestations. Previous studies have indicated that ADS is associated with specific risk factors and symptom profiles, including dysmenorrhea, dyspareunia, and increased menstrual bleeding (Bourdon et al., 2021a; García-Solares et al., 2018). ADS-related chronic pelvic pain and dysmenorrhea severely impair women’s quality of life. A recent study found that dysmenorrhea is negatively correlated with cognitive function (Atlihan et al., 2024), suggesting that dysmenorrhea is not merely a local symptom but is also linked to broader systemic and cognitive changes. Prior research has shown that gut *Roseburia* plays a role in chronic pain and neuropathic pain (Goudman et al., 2024; Lan et al., 2024). By producing SCFAs, it influences the surrounding microbial environment and the host immune system (Steinmeyer et al., 2015), thereby contributing to the regulation of intestinal inflammation and immune maturation (Vacca et al., 2020; Kasahara et al., 2018). The genus *Roseburia* in the gut microbiota may be potentially associated with pain in patients with ADS. *Dialister* is a Gram-negative, anaerobic bacillus belonging to the phylum *Firmicutes*, which can induce host inflammatory responses and insulin resistance through the release of LPS (Mena-Vázquez et al., 2023). *Dialister* was found to be associated with fertility in a study of cervical microbiota in endometriosis (Chang et al., 2022). Research on the endometrial microbiome has also revealed that women who have repeated implantation failure combined with endometriosis have increased *Dialister* abundance (Ono et al., 2024). The present research also found that *Dialister* was positively associated with gravida and parity in ADS. This finding also suggests a possible correlation between *Dialister* and fertility. *Lachnoclostridium* generates short-chain fatty acids that are beneficial for gut homeostasis maintenance. Our results indicate that in ADS, gravida and parity were negatively correlated with *Lachnoclostridium*. In patients with chronic renal disease, *Lachnoclostridium* is negatively correlated with a reduction in 24-h proteinuria (Dong et al., 2024). However, there are no reports on the relationship between *Dialister* and *Lachnoclostridium* with ADS. In previous studies, *Akkermansia* has been found to be associated with sex hormone balance (Liu et al., 2017; He et al., 2020). In fecal virome transplantation-treated mice, the abundance of *Akkermansia* was significantly increased, accompanied by an improvement in fertility (Rasmussen et al., 2023). This study revealed that *Akkermansia* exhibited a significant positive correlation with parity. Combined with previous findings, these results suggest that the abundance of *Akkermansia* in the gut microbiota may confer potential benefits for fertility in individuals with ADS.

There are several strengths of our study, which describe the uniqueness of the intestinal microbiota in ADS patients who did not receive drug therapy. Other similar research has described the characteristics of the gut flora of ADS, but some of the subjects had received hormone therapy (Li J. et al., 2025). However, this study did not measure the hormone levels of sampled patients, which may influence microbiota community composition. Recent studies on hormonal effects on gut microbiota have demonstrated that the use of combined oral contraceptives and levonorgestrel-releasing intrauterine systems shows no significant correlation with gut microbiota composition or diversity in healthy young women (Krog et al., 2022). Conversely, a separate investigation examining the relationship between estrogen and gut microbiota in women revealed a significant association between estradiol levels and both the composition and diversity of gut microbiota communities (Shin et al., 2019). Future research should explore the impact of hormone levels on the microbiota structure in ADS patients, as such investigations would hold substantial value for understanding the etiological mechanisms and therapeutic approaches for ADS. Furthermore, current studies examining the correlation between clinical features of ADS and microbiota remain relatively scarce. Currently, few studies have examined the relationship between the clinical features of ADS and the gut microbiota. This study investigated the association between intestinal flora and clinical characteristics in ADS patients. These findings offer a new microbiome-based perspective on the symptom burden and clinical heterogeneity observed in ADS.

On the other hand, we acknowledge certain limitations in this study. First, the relatively small sample size may constrain statistical power. The study exclusively included a Chinese population, which may limit the geographical generalizability of the findings. Future studies should expand the sample size and adopt a multicenter approach to enhance the representativeness and robustness of the conclusions. Second, detailed dietary habits, lifestyle factors, and environmental exposures were not quantitatively assessed, and menstrual cycle phases were not controlled, variables that could influence the gut microbiota. Nevertheless, all participants were residents of northern China and did not report any specific dietary preferences, suggesting a relatively homogeneous dietary pattern. This may partially, but not completely, mitigate the confounding effects of these variables. A previous study investigating the association between ADS and the vaginal microbiota found differences between the vaginal microbiomes of patients with adenomyosis and healthy individuals during menstruation (Pan et al., 2024). Therefore, further exploration of menstrual phase in future studies may contribute to a deeper understanding of this disease. Future research should meticulously document dietary habits, and more rigorously control for confounding factors to enhance the representativeness and robustness of the conclusions. ADS is a heterogeneous disease; however, due to sample size limitations, this study did not perform subtype classification or severity stratification. Different subtypes of ADS may exert differential effects on microbial composition. Previous analyses of gut microbiota structure in patients with intrinsic versus extrinsic ADS revealed no significant differences in alpha diversity, although linear discriminant analysis effect size (LEfSe) analysis identified differential microbial taxa (Valdés-Bango et al., 2024). Precise subtype classification may hold potential value for elucidating distinct pathogenic pathways and mechanisms underlying different ADS subtypes. Finally, as a cross-sectional study, this research has inherent limitations in establishing causal relationships between ADS and gut microbiota. Whereas 16S rRNA gene sequencing can reveal the taxonomic composition of the gut microbiome in ADS patients, it is limited in strain-level identification. This study employed LEfSe analysis to screen for candidate biomarkers associated with ADS. However, the method has statistical limitations, particularly its lack of FDR correction, which raises the risk of false positives. The analysis of clinical relevance is also constrained by the study’s sample size, and the absence of multivariable regression introduces potential confounding bias. Therefore, future validation in larger, independent cohorts is required. The PICRUSt2 algorithm, which relies on conserved genetic markers, can predict differences in metabolic pathways within microbial communities; however, its functional inferences depend on the coverage of reference databases, potentially leading to the omission of unannotated functional pathways. Further integrating shotgun metagenomics and subsequent experimental validation are required to confirm these findings and explore the interactions between ADS and the microbiota. Nonetheless, this study establishes a foundation for future large-scale and in-depth investigations. Although current research has not yet established a causal relationship between the gut microbiota and ADS, it contributes evidence from a microbial perspective. This finding may serve as a critical catalyst for subsequent exploration, and future experimental studies based on this work should investigate its mechanisms in greater depth.

## 5 Conclusion

In summary, this study identified a distinct gut microbiota composition associated with ADS. Correlations between specific microbial taxa and clinical features indicate a possible link between the gut microbiota and this condition. These findings offer new insights into the etiological factors underlying adenomyosis.

## Data Availability

The data presented in this study can be found in the NCBI SRA (https://www.ncbi.nlm.nih.gov/sra), under accession PRJNA1207430.

## Footnotes

1 https://www.arb-silva.de/ for 16S/18S.
