# Supplementary material for: Intestinal microbiota alteration in women with adenomyosis and clinical characteristics correlation
Source: Front Microbiol. 2026 Aug 6;17:1773822. doi: 10.3389/fmicb.2026.1773822 (PMC13490081; doi:10.3389/fmicb.2026.1773822)
Supplement: Supplementary file 1 [file Supplementary_file_1.DOCX]

Supplementary Material

# 1 Supplementary Table

# 1.1 Supplementary Table S1

**Supplementary Table S1**. Number of raw reads, clean reads for all samples

| **Samples** | **raw reads** | **clean reads** |
| --- | --- | --- |
| ADS_01 | 210175 | 189201 |
| ADS_02 | 206495 | 164724 |
| ADS_03 | 89852 | 69680 |
| ADS_04 | 219212 | 178759 |
| ADS_05 | 205932 | 181425 |
| ADS_06 | 106722 | 87328 |
| ADS_07 | 93135 | 74682 |
| ADS_08 | 105925 | 94680 |
| ADS_09 | 104943 | 88089 |
| ADS_10 | 104902 | 89761 |
| ADS_11 | 103483 | 88951 |
| ADS_12 | 104552 | 96021 |
| ADS_13 | 115703 | 109006 |
| ADS_14 | 104391 | 81330 |
| ADS_15 | 105535 | 98978 |
| ADS_16 | 60506 | 50483 |
| ADS_17 | 103282 | 79187 |
| ADS_18 | 108262 | 81484 |
| ADS_19 | 106335 | 96466 |
| ADS_20 | 106637 | 56429 |
| ADS_21 | 115269 | 105866 |
| ADS_22 | 102353 | 88836 |
| ADS_23 | 108485 | 94365 |
| ADS_24 | 102128 | 89224 |
| ADS_25 | 102973 | 89701 |
| ADS_26 | 114430 | 70286 |
| ADS_27 | 104024 | 86937 |
| ADS_28 | 106650 | 88919 |
| ADS_29 | 118510 | 105027 |
| ADS_30 | 105981 | 90902 |
| Control_01 | 194324 | 142469 |
| Control_02 | 102899 | 92895 |
| Control_03 | 109592 | 94134 |
| Control_04 | 106796 | 94779 |
| Control_05 | 103415 | 90709 |
| Control_06 | 208011 | 200716 |
| Control_07 | 218972 | 208507 |
| Control_08 | 110027 | 94212 |
| Control_09 | 103430 | 95480 |
| Control_10 | 224558 | 151329 |
| Control_11 | 102122 | 88480 |
| Control_12 | 102379 | 78436 |
| Control_13 | 121811 | 109225 |
| Control_14 | 84400 | 74304 |
| Control_15 | 105559 | 100717 |
| Control_16 | 72386 | 69844 |
| Control_17 | 58373 | 49940 |
| Control_18 | 119410 | 102302 |
| Control_19 | 105083 | 96802 |
| Control_20 | 105652 | 98241 |
| Control_21 | 105987 | 68649 |
| Control_22 | 102698 | 89275 |
| Control_23 | 105583 | 85537 |
| Control_24 | 104242 | 84563 |
| Control_25 | 104605 | 68358 |
| Control_26 | 105192 | 64209 |
| Control_27 | 104835 | 60069 |
| Control_28 | 102692 | 69334 |
| Total | 6,841,815 | 5,690,242 |
| Average | 117,962.328 | 98,107.621 |

**1.2** **Supplementary Table S2**

**Supplementary Table S2. Alpha diversity index**

| **Sample Name** | **Goods coverage** | **observed_features** | **chao1** | **shannon** | **simpson** |
| --- | --- | --- | --- | --- | --- |
| ADS group | 1.000 | 2969 | 3196.268 | 7.755 | 0.988 |
| Control group | 1.000 | 3054 | 3301.163 | 7.479 | 0.982 |

# 2 Supplementary Figure

## 2.1 Supplementary Figure S1

**Supplementary Figure S1.** Alpha rarefaction was used to assess the depth of sequencing in the two groups. The red line represents the adenomyosis (ADS) group and the blue line represents the control group.
